# Three Antifungal Proteins From *Penicillium expansum*: Different Patterns of Production and Antifungal Activity

**DOI:** 10.3389/fmicb.2018.02370

**Published:** 2018-10-05

**Authors:** Sandra Garrigues, Mónica Gandía, Laia Castillo, María Coca, Florentine Marx, Jose F. Marcos, Paloma Manzanares

**Affiliations:** ^1^Department of Biotechnology, Instituto de Agroquímica y Tecnología de Alimentos, Consejo Superior de Investigaciones Científicas, Valencia, Spain; ^2^Centre for Research in Agricultural Genomics (CRAG, CSIC-IRTA-UAB-UB), Barcelona, Spain; ^3^Division of Molecular Biology, Biocenter, Innsbruck Medical University, Innsbruck, Austria

**Keywords:** *Penicillium expansum*, PeAfpA, *Penicillium chrysogenum*, Penicillium digitatum, *Botrytis cinerea*, postharvest, crop protection, pathogenic fungi

## Abstract

Antifungal proteins of fungal origin (AFPs) are small, secreted, cationic, and cysteine-rich proteins. Filamentous fungi encode a wide repertoire of AFPs belonging to different phylogenetic classes, which offer a great potential to develop new antifungals for the control of pathogenic fungi. The fungus *Penicillium expansum* is one of the few reported to encode three AFPs each belonging to a different phylogenetic class (A, B, and C). In this work, the production of the putative AFPs from *P. expansum* was evaluated, but only the representative of class A, PeAfpA, was identified in culture supernatants of the native fungus. The biotechnological production of PeAfpB and PeAfpC was achieved in *Penicillium chrysogenum* with the *P. chrysogenum*-based expression cassette, which had been proved to work efficiently for the production of other related AFPs in filamentous fungi. Western blot analyses confirmed that *P. expansum* only produces PeAfpA naturally, whereas PeAfpB and PeAfpC could not be detected. From the three AFPs from *P. expansum*, PeAfpA showed the highest antifungal activity against all fungi tested, including plant and human pathogens. *P*. *expansum* was also sensitive to its self-AFPs PeAfpA and PeAfpB. PeAfpB showed moderate antifungal activity against filamentous fungi, whereas no activity could be attributed to PeAfpC at the conditions tested. Importantly, none of the PeAFPs showed hemolytic activity. Finally, PeAfpA was demonstrated to efficiently protect against fungal infections caused by *Botrytis cinerea* in tomato leaves and *Penicillium digitatum* in oranges. The strong antifungal potency of PeAfpA, together with the lack of cytotoxicity, and significant *in vivo* protection against phytopathogenic fungi that cause postharvest decay and plant diseases, make PeAfpA a promising alternative compound for application in agriculture, but also in medicine or food preservation.

## Introduction

Fungal infections are an emerging worldwide threat to animal, human, and wildlife health (Fisher et al., 2012; Meyer et al., 2016). In medicine and agriculture, control of pathogenic fungi represents a serious challenge due to the increasing number of immunocompromised patients and the emergence of antifungal resistant strains. Accordingly, new antifungal strategies are needed, and current interests are focused on novel antifungal agents with properties and mechanisms of action different from existing ones. Ideally, newly developed antimycotics should also combine major aspects such as sustainability, high efficacy, limited toxicity, and low costs of production (Marx et al., 2008; Meyer, 2008).

Antifungal proteins (AFPs) secreted by filamentous fungi meet the desired characteristics to fight fungal contaminations and infections. AFPs are small, cationic, cysteine-rich proteins highly stable to pH, high temperatures, and proteolysis, and exhibit broad antifungal spectra and different mechanisms of action against opportunistic human, animal, plant, and foodborne pathogenic filamentous fungi (Marx et al., 2008; Hegedüs and Marx, 2013; Delgado et al., 2016). AFPs are coded with a signal peptide (SP) at the N-termini that includes a pre-sequence involved in AFP secretion to the extracellular space, and a pro-sequence, whose function is still controversial although it is assumed that might be involved in maintaining AFPs in an inactive form (Marx et al., 1995).

As shown by genome mining, fungi have a complex repertoire of AFP-like sequences, which are grouped in three major classes A, B, and C (Garrigues et al., 2016). Noteworthy, filamentous fungi genomes encode more than one AFP from different classes. The *Penicillium chrysogenum* genome harbors three genes that code for AFPs belonging to each of three different classes while *Penicillium digitatum* has only one AFP in its genome (class B). The genome of *Neosartorya fischeri* encodes two AFPs (classes A and C) but recently a new AFP has been characterized, which seems to be the first member of a fourth class (Tóth et al., 2016).

As new AFPs are being experimentally identified, differences regarding production, biological function, mode of action and antifungal spectrum are observed. Nowadays, the antifungal activity of at least one representative of all AFP classes has been experimentally demonstrated, and lots of efforts are being made to further examine these proteins. Class A includes those AFPs described firstly, such as PAF from *P. chrysogenum* (Marx et al., 1995) and AFP from *Aspergillus giganteus* (Nakaya et al., 1990; Wnendt et al., 1994; Campos-Olivas et al., 1995; Lacadena et al., 1995) which have been deeply characterized (Meyer, 2008; Hegedüs and Marx, 2013). The first reported class B AFP was Anafp from *Aspergillus niger* (Lee et al., 1999) and currently representatives of class B also include those from *P. chrysogenum* (Delgado et al., 2015; Huber et al., 2018), *P. digitatum* (Garrigues et al., 2017), and *Monascus pilosus* (Tu et al., 2016). Only the antifungal activity of two class C representatives, the BP protein from *Penicillium brevicompactum* (Seibold et al., 2011) and the Pc-Arctin from *P. chrysogenum* (Chen et al., 2013), has been reported.

Some AFP-like proteins are yet uncharacterized, including those from the phytophatogenic fungus *Penicillium expansum*, whose genome contains three genes that code for three different AFP-like proteins, one of each class (Garrigues et al., 2016). Whether the distinct AFP-like proteins within a given fungus are differentially produced, perform different biological functions, or have different antifungal profiles and mode of action is still unknown, and *P. expansum* represents an opportunity to address these issues. In this study, the production of the putative AFPs from *P. expansum* was evaluated, and their antifungal activity demonstrated and described. Only the representative of class A, PeAfpA, was identified in culture supernatants of the native fungus whereas an heterologous expression system in *P. chrysogenum* allowed the production of PeAfpB and PeAfpC. Native and recombinant AFPs have been successfully purified and their characterization showed distinctive antifungal profiles.

## Materials and Methods

### Strains, Media, and Growth Conditions

Fungal strains used in this study were *P. expansum* CECT 20906 (CMP-1) (Ballester et al., 2015), *P. chrysogenum* wild type strain Q176, and *P. chrysogenum* Δ*paf* strain (Hegedüs et al., 2011), which was used as parental strain for fungal transformation. For the antimicrobial assays the following fungal strains were used, (i) filamentous fungi: *P. digitatum* CECT 20796, *Botrytis cinerea* CECT 2100, *Fusarium oxysporum* 4287, *Penicillium italicum* CECT 2294, *A. niger* CBS120.49, *Magnaporthe oryzae* PR9, *Gibberella moniliformis* CECT 2987, *Aspergillus flavus* CECT 20802, *Trichophyton rubrum* CECT 2794, and *Arthroderma vanbreuseghemii* CECT 2958; (ii) yeasts: *Saccharomyces cerevisiae* BY4741, *Candida albicans* CECT 1394*, Candida glabrata* CECT 1448, and *Candida parapsilosis* CECT 1449. Filamentous fungi were cultured on Potato Dextrose Agar (PDA; Difco-BD Diagnostics, Sparks, MD, United States) plates for 7–10 days at 25°C except *A. vanbreuseghemii*, which was grown at 28°C. Yeasts were grown in Glucose Peptone Yeast extract Agar (GPYA) plates at 25°C except *S. cerevisiae*, which was grown at 30°C.

For transformation, vectors were propagated in *Escherichia coli* JM109 grown in Luria Bertani (LB) medium supplemented with 100 μg/mL ampicillin or 75 μg/mL kanamycin. *P. chrysogenum* Δ*paf* was firstly grown in *P. chrysogenum* minimal medium (PcMM) agar (Sonderegger et al., 2016) supplemented with 200 μg/mL nourseotricin for 7 days at 25°C. Conidia were subsequently harvested with a solution containing 0.9% NaCl and 0.01% Tween 80, and were grown in *Aspergillus* complete medium (Sonderegger et al., 2016) for 36 h at 25°C with shaking. Transformants were grown on PcMM plates supplemented with 1 μg/mL pyrithiamine hydrobromide (Sigma-Aldrich, St. Louis, MO, United States). To analyze the growth of the *P. chrysogenum* transformant strains in solid media, 5 μL of conidial suspension (5 × 10^4^ conida/mL) were placed on the center of PDA and PcMM plates, and the colony diameter was monitored daily from 3 to 12 days. For protein production, 200 mL of Potato Dextrose Broth (PDB; Difco-BD Diagnostics) or PcMM were inoculated with a final concentration of 10^6^ conidia/mL of either *P. expansum* CMP-1 or *P. chrysogenum* transformant strains and were incubated for 10 or 4 days, respectively.

### Protein Sequences and Structure Prediction

Sequences from the three different *Peafp* genes and the corresponding amino acid sequences were identified through BLAST searchers that were conducted at the National Center for Biotechnology Information (NCBI) server^1^ (Ballester et al., 2015; Garrigues et al., 2016). Multiple sequence alignments were performed with the Clustal Omega algorithm^2^, using the mature protein sequences without their SP. The I-TASSER software^3^ (Yang et al., 2014) was used to predict the three dimensional (3D) structure of the *P. expansum* AfpA, AfpB, and AfpC, using the *P. chrysogenum* antifungal proteins PAF and PAFB, and the *P. brevicompactum* bubble protein as templates, respectively (Protein Data Bank ID 2MHV, 2NC2, and 1UOY). Models obtained were refined using the ModRefiner software tool^4^ (Xu and Zhang, 2011) and validated by RAMPAGE^5^ (Lovell et al., 2003) to ensure that all amino acids were located inside the favored and energetically allowed regions according to the Ramachandran Plot.

The theoretical molecular weight (MW) and isoelectric point (pI) of the mature proteins were examined with the Compute pI/MW and ProtParam tools of the ExPASy Proteomics Server^6^. All 3D models were visualized by UCSF Chimera software (Pettersen et al., 2004).

### Vector Constructions and *P. chrysogenum* Transformant Strains Generation

Nucleotide sequences of *afpA*, *afpB*, and *afpC* genes were PCR amplified from *P. expansum* CMP-1 genomic DNA, whereas the *paf* gene promoter, SP-pro, and terminator sequences were obtained from the vector pSK275*paf* (Sonderegger et al., 2016). All PCR procedures were performed using AccuPrime High-Fidelity polymerase (Invitrogen, Eugene, OR, United States), and the resulting DNA constructs were purified using High Pure PCR product Purification Kit (Roche, Mannheim, Germany), and verified by Sanger sequencing. Specific primers used for genetic amplification and vector generation are listed in **Supplementary Table S1**. The three different DNA constructions were generated by fusion PCR (Szewczyk et al., 2007) and cloned into the pGEM-T^®^ Easy vector system (Promega, Madison, WI, United States), from where they were excised using two internal restriction sites *Bsp*OI and *Not*I, and subsequently inserted into the previously digested vector pSK275*paf* (pSK275_*PeafpA*, pSK275_*PeafpB*, and pSK275_*PeafpC*) containing the pyrithiamine hydrobromide resistant cassette as positive selection marker.

For the protein production of *P. expansum* AfpA, AfpB, and AfpC in *P. chrysogenum*, the deletion strain Δ*paf* was used as recipient for the plasmids pSK275_*PeafpA*, pSK275_*PeafpB*, and pSK275_*PeafpC*. Protoplast transformation was performed as previously described (Cantoral et al., 1987; Kolar et al., 1988), using 15 μg of *Sma*I linearized plasmids per transformation. Transformant strains were single spored four times on PcMM agar plates supplemented with 1 μg/mL pyrithiamine hydrobromide (Sigma-Aldrich). Positive transformants were confirmed by PCR amplification of genomic DNA (**Supplementary Table S1** and **Supplementary Figure S1**).

### Protein Production and Purification

The *P. digitatum* AfpB was produced and purified as previously described (Hernanz-Koers et al., 2018). PeAfpA was purified from a 10-day PcMM supernatant of *P. expansum* CMP-1 strain. PeAfpB and PeAfpC were purified from supernatants of *P. chrysogenum* transformant strains growing in PcMM for 72–96 h. Cell-free supernatant containing PeAfpA was dialyzed (2 K MWCO, Sigma-Aldrich) against 20 mM phosphate buffer pH 6.6, and supernatants containing PeAfpB and PeAfpC were dialyzed against 20 mM acetate buffer pH 5.4. Dialyzed solutions were applied to an AKTA Purifier system equipped with 6 mL RESOURCE^TM^ S column (GE Healthcare Life Sciences, Little Chalfont, United Kingdom) equilibrated in the corresponding buffer. Proteins were eluted applying a linear gradient from 0 to 1 M NaCl in the same buffer.

Protein containing fractions were pooled, dialyzed against Milli-Q water, and lyophilized. Protein concentrations were determined by spectrophotometry (A_280_) considering their molar extinction coefficients (𝜀_280_ = 0.64 for PeAfpA, and 𝜀_280_ = 0.67 for PeAfpB and PeAfpC). The purity was checked by SDS-PAGE (Laemmli, 1970) using SDS-16% polyacrylamide gels calibrated with prestained protein size-standard SeeBlue^®^ (Thermo Fisher Scientific, Waltham, MA, United States) and Coomassie blue stained.

### Matrix-Assisted Laser Desorption/Ionization–Time-of-Flight Mass Spectrometry (MALDI-TOF MS)

Analyses were performed in the proteomics facility of SCSIE University of Valencia (Spain). The mass of the purified proteins was analyzed on a 5800 MALDI-TOF/TOF (AB Sciex, Framingham, MA, United States) in positive linear mode (1500 shots every position) in a range of 2000–20,000 m/z. For protein identification by peptide mass fingerprinting (PMF), samples were subjected to trypsin digestion and the resulting mixtures analyzed on a 5800 MALDI-TOF/TOF in positive reflectron mode (3000 shots every position). Five of the most intense precursors (according to the threshold criteria: minimum signal-to-noise: 10, minimum cluster area: 500, maximum precursor gap: 200 ppm, maximum fraction gap: 4) were selected for every position for the MS/MS analysis. MS/MS data was acquired using the default 1 kV MS/MS method. The MS and MS/MS information was sent to MASCOT via the Protein Pilot (AB Sciex).

### Antibody Generation and Western Blot

For PeAFPs detection, rabbit polyclonal antibodies were generated as previously described (Mercader et al., 2017) with minor modifications. Procedures for animal immunization were approved by the Ethics Committee of the University of Valencia (Spain) for Animal Experimentation and Welfare (project 2016/VSC/PEA/00136). Animal manipulation was performed according to Spanish and European laws and guidelines concerning the protection of animals used for scientific purposes (RD 1201/2005, Law 32/2007, and European Directive 2010/63/EU). Briefly, two white rabbits of around 2 kg were subcutaneously immunized with 300 μg of each PeAFP in a 1:1 emulsion of phosphate buffer solution and Freund’s adjuvant (Sigma-Aldrich; complete for the first immunization, and incomplete for further boosts). The immunogen was given at least 4 times with intervals of 21 ± 1 days. Blood was taken 10 days after the final injection and it was allowed to coagulate overnight at 4°C. The antibody-containing sera were separated by centrifugation (270 ×*g*, 15 min) and antibodies were precipitated twice with 1 volume of saturated ammonium sulfate solution. Precipitated antisera were stored at 4°C until use.

Total proteins from supernatants and purified AFPs were separated by SDS-16% polyacrylamide gels and transferred to Amersham Protran 0.20 μm NC nitrocellulose transfer membrane (GE Healthcare Life Sciences). Protein detection was accomplished using anti-PeAfpA, anti-PeAfpB, and anti-PeAfpC antibodies diluted 1:2500 for PeAfpA and PeAfpC, and 1:1500 for PeAfpB. As secondary antibody, 1:20,000 dilution of ECL NA934 horseradish peroxidase donkey anti-rabbit (GE Healthcare) was used and chemiluminescent detection was performed with ECL^TM^ Select Western blotting detection reagent (GE Healthcare Life Sciences) using a LAS-1000 instrument (Fujifilm, Tokyo, Japan). The experiments were repeated at least twice.

### Antimicrobial Activity Assays

Growth inhibition assays were performed in 96-well, flat-bottom microtiter plates (Nunc, Roskilde, Denmark) as previously described (Garrigues et al., 2017) with minor modifications. Briefly, 50 μL of fungal conidia (5 × 10^4^ conidia/mL) or yeast cells (2.5 × 10^5^ cells/mL) in 10% PDB containing 0.02% (w/v) chloramphenicol to avoid bacteria contamination were mixed with 50 μL of twofold concentrated proteins from serial twofold dilutions (final concentration 200 μg/mL). Plates were statically incubated for 48 h at 25°C in case of yeasts (*S. cerevisiae* at 30°C), and 72 h at 25°C for filamentous fungi (*A. vanbreuseghemii* at 28°C) except dermatophytes which were incubated for 120 h. Growth was determined every 2 and 24 h, respectively, by measuring the optical density (OD) at 600 nm using FLUOstar Omega plate spectrophotometer (BMG labtech, Orlenberg, Germany), and the OD_600_ mean and standard deviation (SD) between three replicates were calculated. Dose-response curves were generated from measurements after 48 h in yeasts, and 72 h in filamentous fungi (120 h in dermatophytes). These experiments were repeated at least twice. Minimum inhibitory concentration (MIC) is defined as the protein concentration that completely inhibited growth in all the experiments performed.

### Hemolytic Activity Assays

The hemolytic activity of the three PeAFPs was determined in a 96 round-bottom microtiter plate (Nunc) on 1:4 diluted rabbit red blood cells (RBCs) as described (Helmerhorst et al., 1999; Muñoz et al., 2006) with minor modifications. RBCs were harvested by slow centrifugation (100 ×*g*, 15 min) and washed at least three times in 35 mM phosphate buffered saline (PBS, 150 mM NaCl, pH 7) or phosphate buffer glucose (PBG, 250 mM glucose as osmoprotectant). One hundred microliters of twofold protein concentration were mixed with 100 μL of RBCs in triplicate. Plates were incubated for 1 h at 37°C and subsequently centrifuged (300 ×*g*, 5 min). Eighty microliters were transferred to a new microtiter plate and the absorbance was measured at 415 nm (FLUOstar Omega, BMG labtech). Absence of hemolysis and 100% hemolysis were determined in controls with a mixture of PBS or PBG, and 0.1% Triton X-100, respectively. The hemolytic activity was calculated as the percentage of total hemoglobin released compared with that released by incubation with 0.1% Triton X-100.

### Protection Assays Against Fungal Infection Caused by *P. digitatum* in Citrus Fruits

For protection assays, three replicates of five untreated, freshly harvested orange fruits (*Citrus sinensis* L. Osbeck cv. Navelina) were inoculated at four wounds around the equator with 5 μL of a *P. digitatum* conidial suspension (10^4^ conidia/mL) that were pre-incubated for 24 h with different concentrations of PeAfpA and *P. digitatum* AfpB (0.15, 1.5, and 15 μM). Orange fruits were stored at 20°C and 90% relative humidity. The diameter of infection in each wound was measured daily for infection symptoms on consecutive days post inoculation (dpi). Statistical analyses were performed using STATGRAPHICS Centurion 16.7.17. Fisher’s minimum significant difference (LSD) procedure was performed to discriminate between means of % of infected wounds in each treatment with respect to the untreated control at each particular dpi with a 95% confidence.

### Protection Assays Against Fungal Infection Caused by *B. cinerea* in Tomato Leaves

Tomato leaves (*Solanum lycopersicum* cv. Marmande) from 21-days old plants grown at 22°C with 16 h light/8 h dark photoperiod were locally inoculated with conidial suspension of *B. cinerea* alone or in the presence of increasing amounts of AfpB from *P. digitatum* or PeAfpA from *P. expansum*. For this, two drops of 20 μL of the conidial suspension (5 × 10^5^ conida/mL) together with the appropriate concentration of each AFP (1, 5, and 10 μM) were applied onto leaf surfaces. Sterile water was used for the negative control. The plants were maintained with high humidity and the progression of symptoms was measured daily. Leaf damage was quantified by image analysis using the Fiji ImageJ2 package (Schindelin et al., 2012). Statistical analyses were performed using Free Statistics Software, Office for Research Development and Education, version 1.2.1 (Wessa, 2018) to calculate the ANOVA and Tukey’s HSD test.

## Results

### *P. expansum* Encodes up to Three Distinct AFPs From Different Classes but Only Secretes AFP From Class A

**FIGURE 1:**
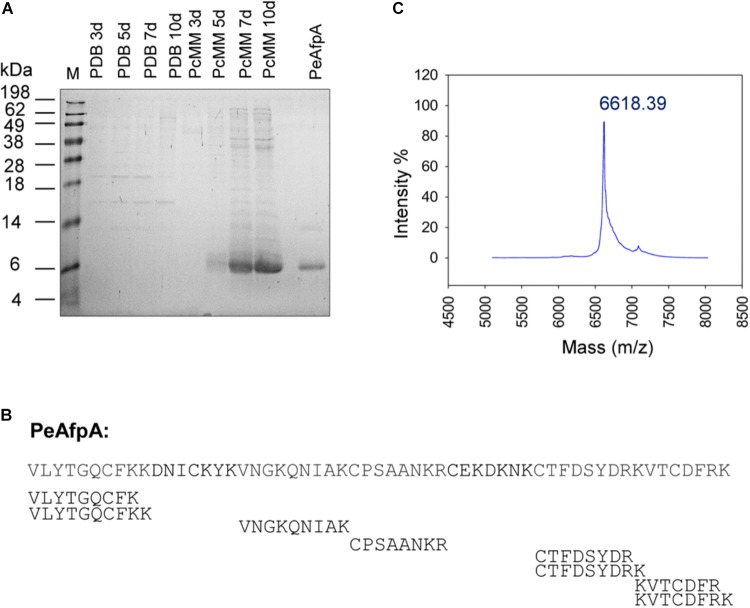
Production and identification of antifungal proteins (AFPs) in *P. expansum* wild-type strain. **(A)** SDS-PAGE of 10 μL of 10× supernatants of *P. expansum* grown in rich medium (PDB) and Minimal Medium (PcMM) for 3, 5, 7 and 10 days and 2 μg of pure PeAfpA. M: SeeBlue^®^ Pre-stained protein standard. **(B)** Peptide mass fingerprinting (PMF) of the class A AFP from *P. expansum* PeAfpA corresponding to the predominant 6 kDa band found in PcMM supernatants. Peptides obtained by PMF covered 78% of PeAfpA primary sequence. **(C)** MALDI-TOF MS analysis. MS shows the isotopic average molecular mass (m/z) of pure PeAfpA produced in *P. expansum.*

In order to detect and isolate any of the three putative *P. expansum* AFPs, called PeAfpA, PeAfpB, and PeAfpC, from culture supernatants, the fungus was grown in either PDB or PcMM growth media, and time-course supernatants were analyzed by SDS-PAGE (**Figure 1A**). *In silico* studies predicted molecular masses of 6.64, 6.57, and 8.12 kDa and pI values of 9.5, 7.6, and 7.7 for PeAfpA, PeAfpB, and PeAfpC, respectively. The largest amount of proteins was detected in PcMM supernatants, from which a protein band of apparent molecular mass of approximately 6 kDa was observed from day 5 till day 10 of growth. No band around 6 kDa was detected in PDB supernatants. To identify the putative PeAFPs produced in PcMM, PMF from an in-gel digestion of the 6 kDa band was performed. A Mascot database search resulted in a statistically significant hit for PeAfpA (score 125; *E*-value 5.8e^-11^) with a sequence coverage of 78% (**Figure 1B**).

According to its predicted chemical properties, PeAfpA purification was achieved from a 10 days *P. expansum* PcMM supernatant by one-step cation-exchange chromatography with yields of 125 mg/L. The protein eluted as a single broad chromatography peak at 0.1–0.5 M NaCl, and SDS-PAGE (**Figure 1A**) and MALDI-TOF MS analyses (**Figure 1C**) revealed a single protein with a molecular mass of 6619.81 Da, which was very similar to that obtained by our previous *in silico* calculations.

### Recombinant Production of PeAFPs in *P. chrysogenum*

**FIGURE 2:**
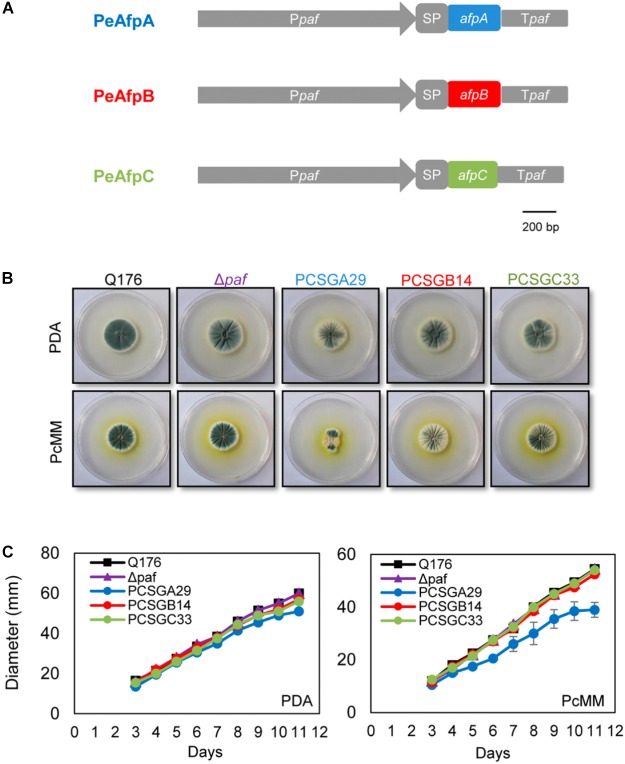
Phenotypical characterization of the *P. chrysogenum* transformant strains producing recombinant PeAFPs. **(A)** Schematic representation of the expression systems used to produce proteins PeAfpA (blue), PeAfpB (red), and PeAfpC (green) in *P. chrysogenum.* In gray: *paf* promoter (P*paf*), *paf* signal peptide (SP), and *paf* terminator (T*paf*). **(B)** Colony morphology of *P. chrysogenum* PeAfpA producer strain (PCSGA29), PeAfpB producer strain (PCSGB14), and PeAfpC producer strain (PCSGC33) compared to the wild type Q176 and the parental strain Δ*paf* after 5 days of growth on PDA and PcMM plates. **(C)** Growth in solid PDA and PcMM determined by the colony diameter from 3 to 11 days of growth at 25°C. Plotted data are mean values ± SD of triplicate samples.

Since only PeAfpA was detected and isolated from the *P. expansum* culture supernatants, we used the *P. chrysogenum*-based expression cassette (Sonderegger et al., 2016; Garrigues et al., 2017) to produce the other two undetected AFPs from *P. expansum* PeAfpB and PeAfpC in *P. chrysogenum* under the regulation of the strong *paf* promoter and terminator sequences (**Figure 2A**). In addition, the PeAfpA production in *P. chrysogenum* was addressed as an internal control. Several positive transformants were obtained and evaluated for protein production in the case of proteins PeAfpB and PeAfpC, and one clone from each with the highest recombinant protein production was selected for further characterization. The selected producer strains were PCSGB14 for PeAfpB and PCSGC33 for PeAfpC. On the contrary, only one positive PeAfpA producer clone, PCSGA29, was obtained. The growth in solid medium of the selected transformants, the reference strain *P. chrysogenum* Q176 and the parental *P. chrysogenum* strain used for transformation (Δ*paf*) are shown in **Figures 2B,C**. The growth of PeAfpB and PeAfpC transformants was indistinguishable from those of the control strains independently of the medium used. In contrast, the PeAfpA transformant showed a significant reduction of colony diameter, more pronounced in PcMM plates, and a drastic defect in conidia production (data not shown). Moreover, the transformant produced little amounts of PeAfpA, which hindered its use for purification of this recombinant protein.

**FIGURE 3:**
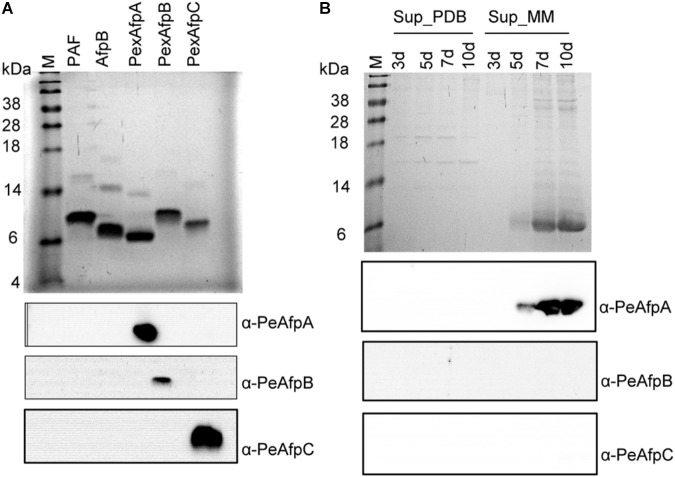
Western blot analyses of pure PeAFPs and growth supernatants of *P. expansum.* **(A)** SDS-PAGE (Top) and western blot analyses (Bottom) of pure PeAfpA, PeAfpB, and PeAfpC (2 μg loaded per lane). Two micrograms of proteins PAF from *P. chrysogenum* and AfpB from *P. digitatum* were added as controls to test cross-reactivity among PeAFPs antibodies. Immunoblot analyses of these samples were performed using specific anti-PeAfpA, anti-PeAfpB, and anti-PeAfpC antibodies generated in this work. **(B)** SDS-PAGE (Top) and Western blot analyses (Bottom) of *P. expansum* culture supernatants (10 μL of 10× supernatants loaded per lane) after 3, 5, 7, and 10 days of growth in PDB and MM. Immunoblot analyses of *P. expansum* supernatants were performed using the three specific PeAFPs antibodies. All SDS-PAGE analyses were visualized by Coomassie blue staining. M: SeeBlue^®^ Pre-stained protein standard.

Selected clones for PeAfpB and PeAfpC production in *P. chrysogenum* were grown in PcMM and, after clearing the culture broth from insoluble matter, the proteins in the supernatants were purified by one-step cation-exchange chromatography. Optimal production was achieved after 72 h with yields of 32 mg/L for PeAfpB and 62 mg/L for PeAfpC. PeAfpB eluted as a broad chromatography peak between 0.15 and 0.3 M NaCl while PeAfpC eluted as a sharp single peak at 0.075 M NaCl. SDS-PAGE analysis revealed a protein band in both protein samples, having apparent molecular masses higher than 6 kDa. PeAfpB showed less migration than expected from its predicted molecular mass (6.57 kDa) and in comparison to purified PeAfpA (**Figure 3A**, top panel).

Molecular masses of both recombinant proteins were determined by MALDI-TOF MS. Single peaks corresponding to average masses of 6576.07 and 6718.5 Da were detected for PeAfpB and PeAfpC, respectively (**Supplementary Figure S2**). The experimental mass of PeAfpB is consistent with the calculated theoretical mass of the oxidized protein predicted after cleavage from the PAF SP-pro sequence (6572.2 Da), indicating the presence of three intra-molecular disulphide bonds and the absence of other post-translational modifications. By contrast, the average mass detected for PeAfpC was lower than the theoretical mass expected of 8123 Da, suggesting an inappropriate processing. To verify the identity of the recombinant PeAfpC produced in *P. chrysogenum*, PMF analysis of the purified protein was done. A Mascot database search resulted in a statistically significant hit for DUF1962 (protein with domains of unknown function) from *P. expansum* (score 280; *E*-value 7.9e^-21^) with a sequence coverage of 53% (**Supplementary Figure S3**). This protein corresponded to PeAfpC, which in the genomic annotation included an internal insertion of 11 extra amino acids that were theoretically present in the three different *P. expansum* sequenced strains but absent in class C proteins from other fungi (Ballester et al., 2015; Garrigues et al., 2016; **Supplementary Figure S3A**). Our data demonstrate that this insertion is absent in our purified PeAfpC (**Supplementary Figure S3B**). These results indicated that PeAfpC has a theoretical pI of 6.87 and a predicted molecular mass of 6.72 kDa, in accordance to that experimentally determined (6718.5 Da), and similar to that reported for other homologs belonging to the same class.

### Immunodetection Confirmed the Absence of PeAfpB and PeAfpC in *P. expansum* Supernatants

Purified PeAFPs were used to generate polyclonal antibodies. The polyclonal anti-PeAfpA, anti-PeAfpB, and anti-PeAfpC specifically recognized the corresponding purified protein while no cross reactivity among the three proteins was observed (**Figure 3A**, bottom panel). Purified PAF from *P. chrysogenum* and AfpB from *P. digitatum* were also included as representatives of classes A and B proteins, respectively. However, neither the polyclonal anti-PeAfpA recognized PAF nor anti-PeAfpB immunoreacted with *P. digitatum* AfpB (**Figure 3A**, bottom). Specific signals were also detected in the supernatants of the selected PeAFP producer *P. chrysogenum* transformant strains PCSGA29, PCSGB14, and PCSGC33 (**Supplementary Figure S4**).

Polyclonal antibodies were then used to analyze the supernatants of *P. expansum*. In the *P. expansum* supernatants that were initially analyzed by Coomassie blue staining (**Figure 3B**, top panel), neither PeAfpB- nor PeAfpC-specific signals could be immunodetected in either PDB or PcMM culture supernatants. As expected, PcMM supernatants only reacted with the anti-PeAfpA antibody, and no immunoreaction was observed in the PDB culture supernatants (**Figure 3B**, bottom panel), confirming that *P. expansum* only produces PeAfpA naturally in PcMM under the conditions tested.

### PeAFPs Structural Modeling

**FIGURE 4:**
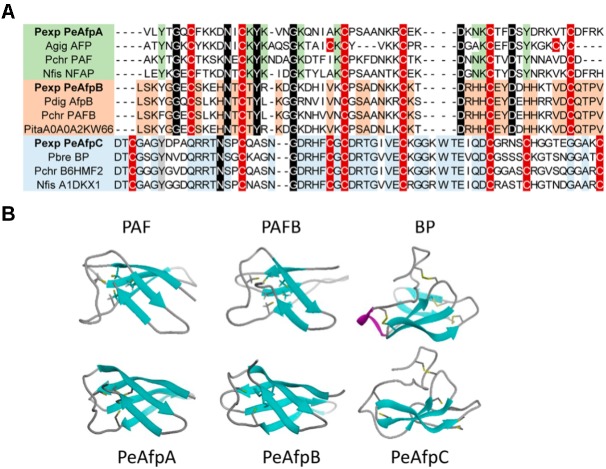
Molecular modeling of *P. expansum* AFPs of classes A, B, and C. **(A)** Amino acid sequence alignment of a selection of AFPs and AFP-like proteins. Proteins belonging to different phylogenetic classes are highlighted in different colors. Proteins belonging to class A are represented in green, while classes B and C are shaded in orange and blue, respectively. Conserved intra-class motifs are shadowed following their color code. Cysteine patterns are shadowed in red. Strongly conserved amino acids between classes are shadowed in black. **(B)** Comparison of the predicted tertiary structure of PeAfpA, PeAfpB, and PeAfpC from *P. expansum* with the three-dimensional structures of the proteins PAF and PAFB from *P. chrysogenum* and BP from *P. brevicompactum* used as templates, respectively.

The 3D structure of mature PeAFPs was predicted by homology modeling using protein PAF (PDB ID 2MHV) (Fizil et al., 2015) and PAFB (PDB ID 2NC2) (Huber et al., 2018) from *P. chrysogenum* and BP protein from *P. brevicompactum* (PDB ID 1UOY) (Olsen et al., 2004) as templates for PeAfpA, PeAfpB, and PeAfpC, respectively (**Figure 4**).

PeAfpA and PeAfpB show 53 and 77% amino acid identity with the *P. chrysogenum* PAF and PAFB, respectively. Tertiary structure of PeAfpA and PeAfpB were very similar to their classes A and B homologs, with five antiparallel β-strands forming a compact β-barrel that would be theoretically stabilized by three disulphide bonds following the abcabc pattern, as described for PAF and PAFB (Váradi et al., 2013; Huber et al., 2018).

PeAfpC shows 74% amino acid identity with the BP protein used as template. However, PeAfpC predicted structure significantly differs from that of the BP. BP contains five antiparallel β-strands and four disulphide bonds connecting the two compacted β-sheets forming a basic accessible shallow funnel that may be relevant to the protein function (Olsen et al., 2004). Furthermore, BP contains a small α-helix structure absent in the other classes of AFPs. On the contrary, PeAfpC is predicted to have partially lost its tertiary structure if compared with BP. PeAfpC only contains three antiparallel β-strands forming a compacted β-sheet, whereas the second β-sheet and α-helix structures present in BP are missing in PeAfpC (**Figure 4**).

### Antimicrobial Activity Assays

The three PeAFPs were tested for their antimicrobial activity toward a selection of filamentous fungi that include the *P. expansum* parental strain and several plant pathogens such as the citrus fruit specific *P. digitatum* and *P. italicum*, the polyphagous *B. cinerea*, the rice blast fungus *M. oryzae*, and the soilborne plant pathogen *F. oxysporum*. Furthermore, the mycotoxin producers *A. flavus* and *G. moniliformis*, and clinically relevant pathogens such as the skin pathogens *T. rubrum* and *A. vanbreuseghemii*, and the opportunistic human pathogens *C. albicans*, *C. glabrata*, and *C. parapsilosis* were also examined. Finally, *S. cerevisiae*, the PAF producer *P. chrysogenum* strain, and a strain from *A. niger* which is particularly sensitive to AFPs were also evaluated.

**FIGURE 5:**
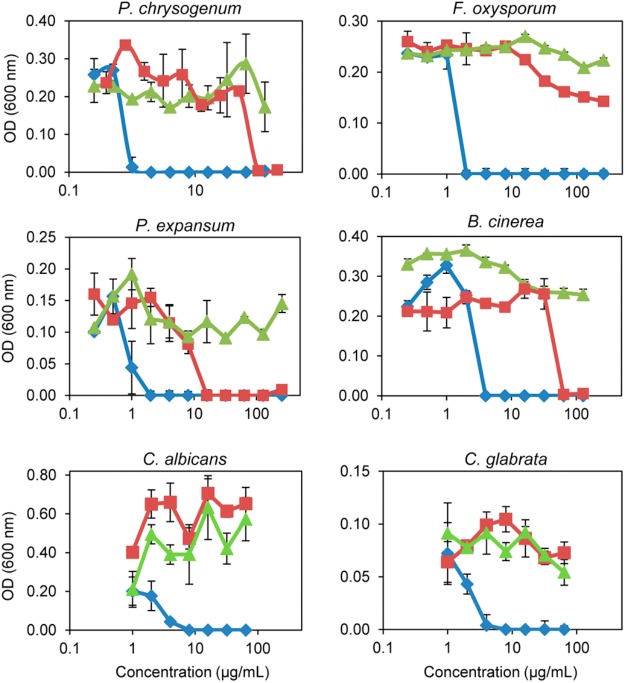
*In vitro* inhibitory activity of the three PeAFPs against filamentous fungi and yeasts. Dose-response curves comparing the antifungal activity of PeAfpA (blue diamonds), PeAfpB (red squares), and PeAfpC (green triangles) against the filamentous fungi *P. chrysogenum*, *F. oxysporum*, *P. expansum*, and *B. cinerea*, and the pathogenic yeast*s C. albicans* and *C. glabrata*. Dose-response curves show mean ± S.D. OD_600_ of triplicate samples after 72 h at 25°C for fungi and 48 h at 28°C for yeast.

Differences in antimicrobial activity were observed among the three PeAFPs (**Table 1** and **Figure 5**). PeAfpA showed high antifungal activity and inhibited the growth of all tested fungi. The minimum inhibitory concentration (MIC) values varied from 1 μg/mL against *P. digitatum* to 16 μg/mL against *M. oryzae*. The *Penicillium* species tested and *A. niger* were more susceptible to PeAfpA, including the producer parental strain *P. expansum*. By contrast, PeAfpC was inactive against all the fungi at the highest concentration tested (200 or 64 μg/mL), while PeAfpB showed a moderate antifungal activity with MIC values ranging from 12 μg/mL against the three phytopathogenic *Penicillium* species to 50 μg/mL against *P. chrysogenum*, *B. cinerea*, and *A. niger*. PeAfpB was not active against either *M. oryzae* or *F. oxysporum* at 200 μg/mL or against *G. moniliformis*, *A. flavus*, or *A. vanbreuseghemii* at 64 μg/mL. PeAfpB was also inactive against yeast species.

**Table 1:** Minimal inhibitory concentration (MIC) values (μg/mL) of PeAFPs against all the fungi tested^1^.

Fungi	PeAfpA	PeAfpB	PeAfpC
*P. expansum*	2	12	>200
*P. digitatum*	1	12	>200
*P. italicum*	2	12	>200
*P. chrysogenum*	2	50	>200
*B. cinerea*	4	50	>200
*M. oryzae*	16	>200	>200
*F. oxysporum*	4	>200	>200
*G. moniliformis*	4	>64	>64
*A. flavus*	4	>64	>64
*A. niger*	2	50	>200
*T. rubrum*	4	32	>64
*A. vanbreuseghemii*	4	>64	>64
*C. albicans*	8	>64	>64
*C. glabrata*	4	>64	>64
*C. parapsilosis*	4	>64	>64
*S. cerevisiae*	4	>64	>64

*^1^MIC values were determined at 72 h for fungi (120 h for dermatophytes) and 48 h for yeasts.*

### PeAFPs Showed No Hemolytic Activity

Hemolytic activity assays are performed in order to determine the cytotoxicity of specific proteins and peptides against eukaryotic cells by their ability to lyse RBCs. The hemolytic activity of the three PeAFPs and of the cytolytic peptide melittin as positive control was determined using a high ionic strength phosphate NaCl buffer (PBS) and also a low ionic strength isotonic glucose phosphate buffer (PBG) (Helmerhorst et al., 1999).

**FIGURE 6:**
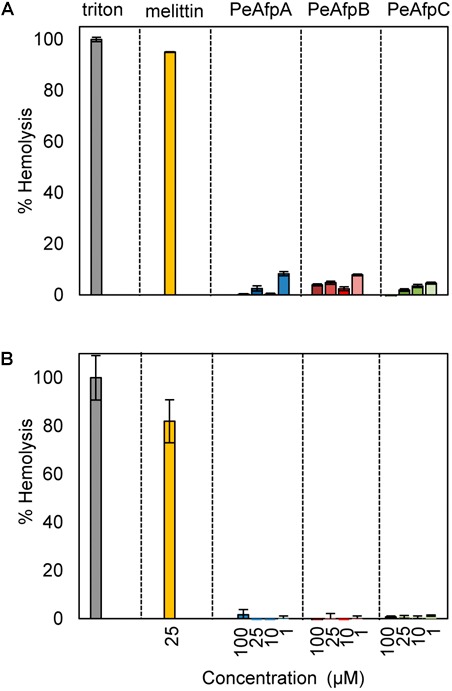
Hemolytic activity of the three AFPs from *P. expansum*. Analyses were conducted in PBS (150 mM NaCl) **(A)**, and in PBG (250 mM glucose) **(B)**. Proteins were used at the concentrations indicated (from 1 to 100 μM). For PeAFPs, 1, 10, 25, and 100 μM correspond to 6.6, 66, 166, and 662 μg/mL for PeAfpA; 6.5, 65, 164, and 657 μg/mL for PeAfpB; and 6.7, 67, 168, and 678 μg/mL for PeAfpC, respectively. The cytolytic peptide melittin (25 μM) was included for comparison. The hemolytic activity is given as the mean ± SD of the percentage of mammal red blood cells (RBCs) hemolysis (three replicates), as compared with the positive control in the presence of the detergent Triton X-100 (regarded as 100% hemolysis).

None of the PeAFPs showed hemolytic activity at any of the concentrations tested (1–100 μM), neither in the presence of NaCl as in PBS (**Figure 6A**) nor glucose (**Figure 6B**), in contrast to the hemolysis caused by melittin at 25 μM.

### PeAfpA Confers Protection Against *P. digitatum* Infection in Orange Fruits

**FIGURE 7:**
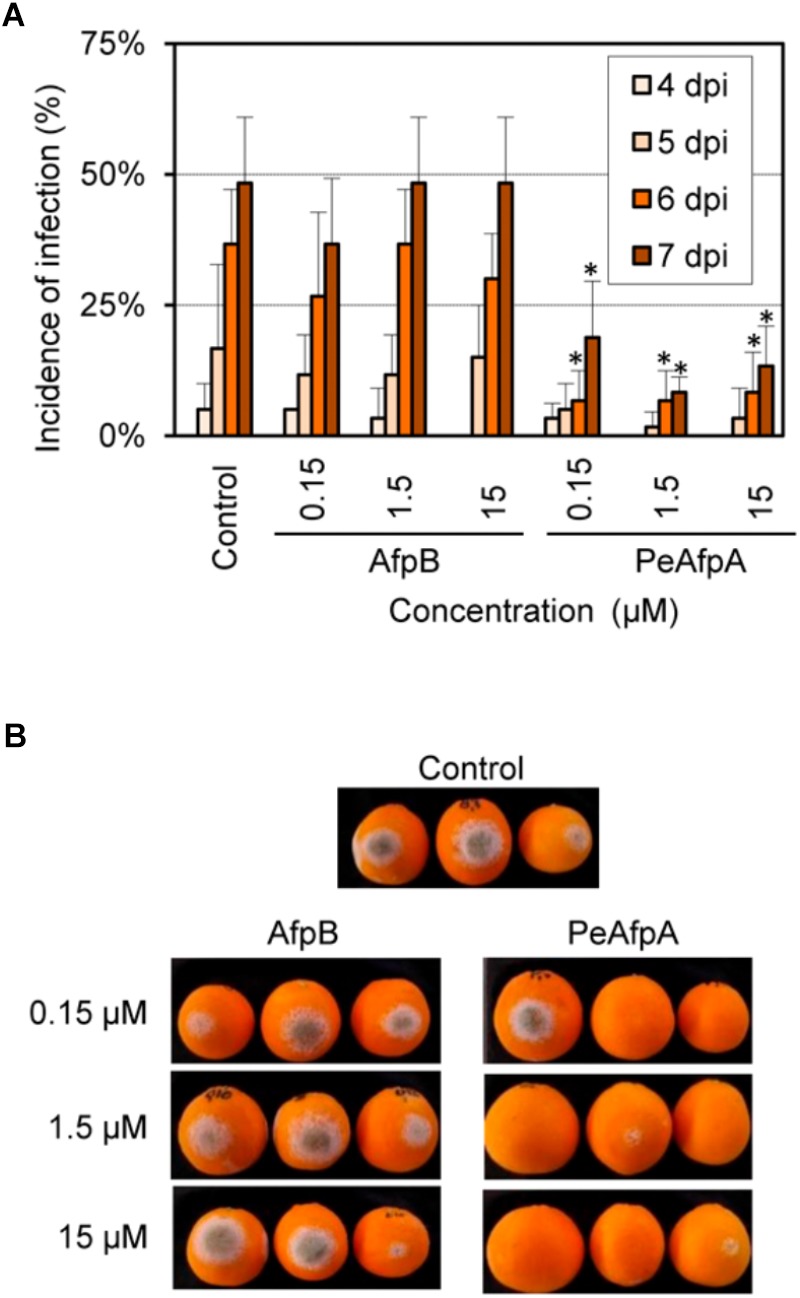
Effect of *P. digitatum* AfpB and *P. expansum* PeAfpA on the infection of citrus fruits caused by *P. digitatum.* **(A)** Incidence of infection of inoculated wounds. Orange fruits were inoculated with 10^4^ conida/mL of *P. digitatum* either alone (Control) or in the presence of 0.15, 1.5, and 15 μM (1, 10, and 100 μg/mL, respectively) of AfpB and PeAfpA. Bars show the mean values of the percentage of infected wounds and SD of three replicates of five oranges at 4, 5, 6, and 7 days post-inoculation (dpi). Asterisks show statistical significance of the infection incidence compared to the control samples at each independent day (Fisher’s LSD test, ^∗^*p* < 0.05). **(B)** Representative images of treated oranges with AfpB and PeAfpA at the indicated concentrations at 7 dpi.

Based on the *in vitro* antimicrobial results, experiments were designed to evaluate PeAfpA ability to control the green mold disease caused by *P. digitatum* infection to citrus fruit. AfpB from *P. digitatum*, which has been previously described as a highly *in vitro* active AFP (Garrigues et al., 2017), was also included as a potential candidate to control green mold. **Figure 7** shows the effects of different concentrations of AfpB and PeAfpA. The latter showed control of experimental *P. digitatum* infections when used at concentrations as low as 0.15 μM at late dpi. Contrarily, AfpB showed no significant protection at any of the concentrations tested (*p* < 0.05).

### PeAfpA Confers Protection Against *B. cinerea* Infection in Tomato Leaves

**FIGURE 8:**
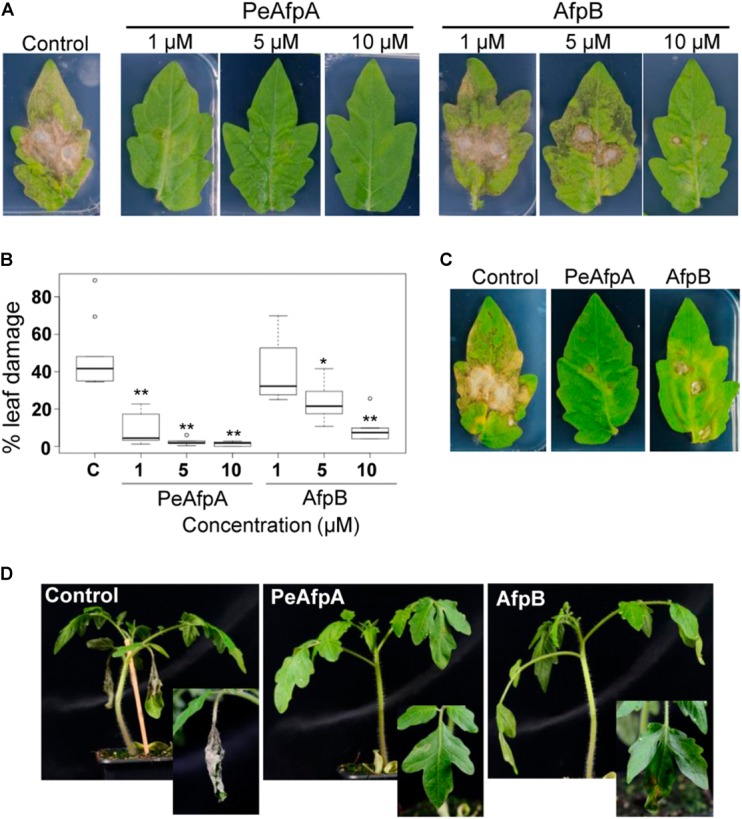
Tomato plants protection against *B. cinerea* infection by PeAfpA and *P. digitatum* AfpB. Cut leaves **(A–C)** or whole tomato plants **(D)** were infected in two different points per leaf with fungal conidia suspensions (5 × 10^5^ conidia/mL) along with water (c, control) or the indicated amount of PeAfpA or AfpB, simultaneously **(A,B,D)** or after 6 h **(C)**. Concentrations of 1, 5, and 10 μM correspond to 6.6, 32, and 66 μg/mL, respectively. Ten micromolar (66 μg/mL) of AFPs were applied in panels **(C,D)**. Pictures were taken at 4 days post inoculation. Graph in panel **(B)** is a box plot of the percentage of leaf damage quantified from at least six leaves per treatment from two independent assays. Asterisks denote statistically significant differences in comparison to control values (ANOVA and Tukey’s HSD test ^∗∗^*p* < 0.001; ^∗^*p* < 0.05).

Experiments were designed to assess the effectiveness of PeAfpA against the infection caused by the polyphagous fungus *B. cinerea in vivo* in a detached leaf assay. Recently we have shown the effectiveness of *P. digitatum* AfpB at a concentration of 10 μM to control *B. cinerea* in tomato leaves (Shi et al., unpublished), and thus AfpB was included here as a positive control and for comparison of antifungal efficacy with PeAfpA. Development of disease symptoms on the detached leaves was monitored visually. Four days after inoculation, lesions were observed in the fungus-infected leaves that had not been treated with proteins (control) (**Figure 8A**). However, lesions were not observed or were significantly smaller when treated with PeAfpA (**Figures 8A,B**). This protective effect was dependent on protein doses, being still effective even at concentrations as low as 1 μM, and greater than the caused by PeAfpB (**Figures 8A,B**). Interestingly, protection afforded by both PeAfpA and AfpB was also effective on established infection foci when proteins were applied 6 h after conidia (**Figure 8C**). This data suggests that both proteins can be used to treat already infected plants. The protective effect of these AFPs was also observed in whole plant assays in which two leaves per plant were inoculated (**Figure 8D**). The control plants showed complete necrosis of the inoculated leaves and mild systemic signs of decay, while Afp-treated plants showed little or no infection symptoms.

## Discussion

In this study, we detail the differential patterns of production of the three AFPs from the phytopathogenic fungus *P. expansum*. PeAfpA, PeAfpB, and PeAfpC are new members of classes A, B, and C, respectively, and here we experimentally characterize their antifungal activity.

Only PeAfpA was detected in culture supernatants of *P. expansum* when grown in MM with sucrose as carbon source while in the nutritionally rich medium PDB (potato infusion + glucose) no protein was observed. The class A member PAF is also abundantly secreted by *P. chrysogenum* but its production depends on the type of carbon source present in the growth medium (Marx et al., 1995). AFP, another representative of class A, was successfully isolated from the culture supernatants of *A. giganteus* when grown in a rich medium based on corn starch and beef extract (Lacadena et al., 1995). Expression studies performed with *afp* and *paf* do not indicate a general pattern for both genes, except that the maximum mRNA and protein yield is reached during the stationary growth phase after 70–90 h of cultivation (Meyer and Stahl, 2002; Marx, 2004). Our time course experiments for protein production showed that PeAfpA was detected in MM *P. expansum* supernatants from day 5, and high yields of the protein (125 mg/L) were reached from 10 day-old supernatants. Thus, cultivation conditions seem to regulate PeAfpA production since the protein was neither detected by Coomassie staining nor by anti-PeAfpA antibodies in PDB supernatants. PeAfpA production at such long incubation times in MM suggests that it might be linked to nutrient limitation as described for PAF and AFP, and that glucose might suppress production (Marx et al., 1995). Remarkably, a given fungal strain might produce different AFPs depending on culture broth as described for *N. fischeri* NRRL 181. The class A NFAP was isolated when the fungus was grown in a complex medium with starch, beef extract, peptone, NaCl and ethanol for 7 days (Kovács et al., 2011) while NFAP2 but no NFAP was isolated from a 7-day old MM supernatant with sucrose as carbon source (Tóth et al., 2016).

PeAfpB and PeAfpC were not detected in any of the conditions tested. Instead, both proteins were produced using a *P. chrysogenum*-based expression system (Sonderegger et al., 2016), and they were purified from supernatants of recombinant *P. chrysogenum* strains. This expression system comprises the strong *paf* gene promoter, the *paf* pre-pro sequence for correct protein processing and secretion, and the *paf* gene terminator (Marx et al., 1995). This system allowed the production of high amounts of several AFPs (Sonderegger et al., 2016, 2017), including *P. digitatum* AfpB and *P. chrysogenum* PAFB which could not be isolated from the supernatants of the corresponding parental strains (Garrigues et al., 2016, 2017; Huber et al., 2018). Here, the heterologous production of PeAfpB and PeAfpC in *P. chrysogenum* resulted in yields of 32 and 62 mg/L, respectively, confirming the suitability of the system as a platform for the production of small cysteine-rich AFPs. Further studies focusing on gene expression patterns will reveal whether the lack of PeAfpB and PeAfpC in the *P. expansum* culture broth results from a strict regulation during fungal growth, similar to the reports of AfpB (Garrigues et al., 2016) and PAFB (Huber et al., 2018), or in contrast, from non-functional or unexpressed genes.

Peptide mass fingerprinting of the recombinant PeAfpC revealed that this protein lacked the 11 amino acid insertion that was predicted by *in silico* annotation of three different sequenced strains of *P. expansum* (Ballester et al., 2015). Genes coding for AFPs from classes A and B have two introns, whereas genes coding for class C AFPs have only one (Garrigues et al., 2016). The predicted insertion within PeAfpC amino acid sequence correlates with an incorrect annotation of the single intron present in the class C AFP encoding gene from *P. expansum*. Thus, PeAfpC is similar to other characterized and putative class C AFPs regarding their size and chemical properties.

Two of the three PeAFPs are effective against filamentous fungi while PeAfpC did not show any antimicrobial activity under the conditions tested. One possible explanation for this different activity patterns could be explained with their distinct physico-chemical properties, especially the positive net charge at pH 7, which would correlate with their ability to bind fungal membranes. PeAfpA, which showed the highest antifungal activity, is a very cationic protein with a pI of 9.47. In contrast, PeAfpB (pI = 7.4) showed a moderate antifungal activity against some of the fungi tested, but not against yeasts, and this protein showed a lower antifungal activity when compared to its class B homolog AfpB from *P. digitatum* (pI = 9.06). On the contrary, PeAfpC (pI = 6.87) was inactive against all fungi and yeasts tested in this work. Only the antifungal activity of two other class C representatives, the BP protein from *P. brevicompactum* (Seibold et al., 2011) and the Pc-Arctin from *P. chrysogenum* (Chen et al., 2013), have been reported. The former showed antifungal activity against *S. cerevisiae* and no other fungal species were evaluated (Seibold et al., 2011), while Pc-Arctin was effective against some plant pathogenic fungi (Chen et al., 2013).

The predicted 3D structure of PeAfpC significantly differs from the one experimentally determined for its class C homolog BP from *P. brevicompactum*. A loss of the three-dimensional organization in the PeAfpC *in silico* predicted structure might explain the loss of its antifungal activity. However, it has been reported that structural features of AFPs are not exclusively responsible for their antifungal activities. This is the case for *P. digitatum* AfpB, where we demonstrated that thermal denaturation did not affect its antifungal activity (Garrigues et al., 2017), or for PAF from *P. chrysogenum*, where the change of a single amino acid did not affect its 3D structure, but resulted in a complete loss of antifungal efficacy (Sonderegger et al., 2017). Recently, the anti-viral activity of some AFPs has been documented for the first time (Huber et al., 2018), suggesting that the properties of AFPs go beyond the traditional antifungal activity. Further structural and functional characterization of PeAfpC is currently in progress.

PeAfpA is the most potent AFP from *P. expansum*. It is highly effective against relevant phytophatogenic fungi that cause postharvest decay and plant diseases. Moreover, we have shown that PeAfpA exerted significant protection against *P. digitatum* in oranges and against *B. cinerea* in tomato leaves. The application of antimicrobial peptides and proteins in postharvest conservation and crop protection has been described (Coca et al., 2004; Marcos et al., 2008). To our knowledge, AFP from *A. giganteus* is the only AFP that has been previously shown to successfully protect plants from fungal infection, albeit at higher protein doses than used in our assays. Similarly to the *in vivo* experiments described here, rice plants were protected from *Magnaporthe grisea* infection by direct application of 10 μM AFP to rice leaves either by drops or spray (Vila et al., 2001) and geranium plants from *B. cinerea* (Moreno et al., 2003). *A. giganteus* AFP at a concentration of 100 μg/mL preincubated with tomato seedlings also prevented the infection of tomato roots by the plant-pathogenic fungus *F. oxysporum* f.sp. *lycopersici* (Theis et al., 2005). Moreover, AFP sprayed on artificially infected-wounded bananas with *Alternaria alternata* was able to partly or totally inhibit the growth of the phytopathogen at concentrations in the range 15–50 μg/mL (Barakat, 2014). For crop protection, strategies based on the heterologous expression of the *A. giganteus afp* encoding gene conferred enhanced resistance to transgenic rice plants against the blast fungus *M. oryzae* (Coca et al., 2004), to transgenic wheat plants against the powdery mildew fungus *Erysiphe graminis* f.sp. *tritici* and the leaf rust fungus *Puccinia recondite* f.sp. *tritici* (Oldach et al., 2001) and to transgenic olive plants against the root infecting fungal pathogen *Rosellinia necatrix* (Narváez et al., 2018).

Our results show that PeAfpA is highly effective in controlling *P. digitatum* and *B. cinerea* infections in citrus and tomato at concentrations as low as 0.15–1 μM. Both fungi have a considerable economic importance. Severe fruit losses due to *Penicillium* decay have an important impact in agriculture, especially decay caused by *P. digitatum*, one of the main postharvest pathogens of citrus fruits. *P. digitatum* specifically infects citrus fruits through peel injuries produced in the field, the packing house or during the fruit commercialization chain, causing the green mold disease (Palou, 2014). By contrast, the impact of *B. cinerea* in many areas is due to its broad host range, causing severe damage, both pre- and postharvest (Dean et al., 2012). Despite the effectiveness of commercial chemical fungicides, concerns about environmental contaminations, the emergence of resistant strains and human health risks associated with fungicide residues lead to the search of new control strategies. Thus, PeAfpA might represent a powerful alternative in the control of phytopathogenic fungi. Moreover, considering the broad *in vitro* antifungal activity of PeAfpA against phytopathogenic fungi and against mycotoxins producers, it seems feasible that the protein may be effective also in other pathosystems not tested in this study. Our results also point to the heterologous expression of *P. expansum afpA* encoding gene in transgenic plants to confer disease resistance. Previously we described a very promising efficacy of the synthetic hexapeptide PAF26 and derivatives in citrus fruit protection (López-Garcia et al., 2003; Muñoz et al., 2007), although the high cost of synthetic peptide production and the failure to produce PAF26 through biotechnology (unpublished data) poses an obvious limit to postharvest applications. By contrast, different expression systems, including the one used here, allow effective AFP-production (Sonderegger et al., 2016; Garrigues et al., 2017; Patiño et al., 2018; Shi et al., unpublished), enabling the use of AFPs in crop and postharvest protection.

*Penicillium digitatum* AfpB identified as an *in vitro* highly active AFP against the own producer fungus (MIC = 3.2 μg/mL) (Garrigues et al., 2017) showed no *in vivo* effect in oranges as it did against *B. cinerea* in tomato leaves (MIC = 12.5 μg/mL). Until recently, it was assumed that AFPs were not active against the producer fungus. However, in addition to *P. digitatum* AfpB, PAFB (Huber et al., 2018) and now PeAfpA are effective toward *P. chrysogenum* and *P. expansum*, respectively. *In vitro* AFP growth inhibition against the own producer fungus is induced adding the protein exogenously to the culture media. Whether *in vivo* activity parallels that observed in *in vitro* tests deserves further studies.

Interestingly PeAfpA is also highly active against human fungal pathogens including dermatophytes (MIC 4 μg/mL), clinically important *Candida* species (MIC values 4–8 μg/mL), and also against mycotoxin-producer fungal strains (MIC 4 μg/mL), suggesting its potential application also in medicine and food preservation. The use of antimicrobial peptides for the prevention and treatment of fungal skin infections like those caused by *T. rubrum* and *A. vanbreuseghemii* has been proposed (López-García et al., 2007). AFPs such as PAF and PAFB from *P. chrysogenum* were active against *T. rubrum* with similar MIC values as that described here for PeAfpA (Huber et al., 2018). Nevertheless, further characterization of AFPs in *in vivo* models are mandatory to confirm the potential of AFPs as novel therapies to treat dermatological diseases. Originally, AFPs were described as highly effective against filamentous fungi but not active against yeasts or bacteria (Marx et al., 2008; Meyer, 2008). However, anti-yeast activity of PAF was recently re-evaluated and its effectiveness against *S. cerevisiae* and *C. albicans* was reported, as well as that of PAFB (Huber et al., 2018). PAFB was the most active against both yeasts species with MIC values similar to those obtained here for PeAfpA. At present, NFAP2 is the most potent anti-yeast AFP described so far, with MIC values in the range of 0.2–1.5 μg/mL (Tóth et al., 2016). Remarkably, this protein, which seems to be the first member of a new, phylogenetically distinct fourth group among AFPs, was ineffective against filamentous fungal isolates whereas the opposite antifungal profile was determined for the class A NFAP (Virágh et al., 2014).

Toxicity of antimicrobials should also be considered for successful application. The toxicity of PeAFPs has been measured as their cytolytic activity against RBCs. The hemolytic activity of the three proteins was negligible in the conditions tested, even in assays conducted at low ionic strength isotonic conditions, which are considered more sensitive for detecting the hemolytic activity of cationic peptides (Helmerhorst et al., 1999). The lack of cytotoxicity was previously reported for PAF (Szappanos et al., 2005; Palicz et al., 2013) and *A. giganteus* AFP (Szappanos et al., 2006), and recently for *P. digitatum* AfpB (Garrigues et al., 2017) and *P. chrysogenum* PAFB (Huber et al., 2018), suggesting that AFPs can be regarded as safe.

## Conclusion

To conclude, the high antifungal efficacy against human and plant pathogens and mycotoxin-producer fungi, together with the protection observed here upon application of PeAfpA for postharvest conservation of orange fruits and plant protection on tomato leaves, suggest that PeAfpA is a promising candidate for crop and postharvest protection and for its application in medicine or food security.

## Author Contributions

MC, FM, JM, and PM conceived and designed the study. PM coordinated the study and prepared the first draft of the manuscript. SG and FM produced AFPs in *P. chrysogenum*. SG and PM produced AFP in *P. expansum*. SG and JM performed antimicrobial experiments and structural modeling. MG and SG performed Western blot analyses and performed hemolytic assays and protection assays in citrus fruits. LC and MC carried out protection assays in tomato plants. All authors read, revised, and approved the final manuscript.

## Conflict of Interest Statement

The authors declare that the research was conducted in the absence of any commercial or financial relationships that could be construed as a potential conflict of interest.

## Footnotes

1 https://www.ncbi.nlm.nih.gov/

2 https://www.ebi.ac.uk/Tools/msa/clustalo/

3 https://zhanglab.ccmb.med.umich.edu/I-TASSER/

4 https://zhanglab.ccmb.med.umich.edu/ModRefiner/

5 http://mordred.bioc.cam.ac.uk/~tilderapper/rampage.php

6 http://web.expasy.org
