# Supplementary material for: Three Antifungal Proteins From Penicillium expansum: Different Patterns of Production and Antifungal Activity
Source: Front Microbiol. 2018 Oct 5;9:2370. doi: 10.3389/fmicb.2018.02370 (PMC6182064; doi:10.3389/fmicb.2018.02370)
Supplement: Supplementary file 1 [file Data_Sheet_1.docx]

Supplementary Material

Three antifungal proteins from *Penicillium expansum*: different patterns of production and antifungal activity

Sandra Garrigues, Mónica Gandía, Laia Castillo, María Coca, Florentine Marx, Jose F. Marcos and Paloma Manzanares*

*** Correspondence:** Paloma Manzanares: pmanz@iata.csic.es

# Supplementary Figures and Tables

## Supplementary Figures


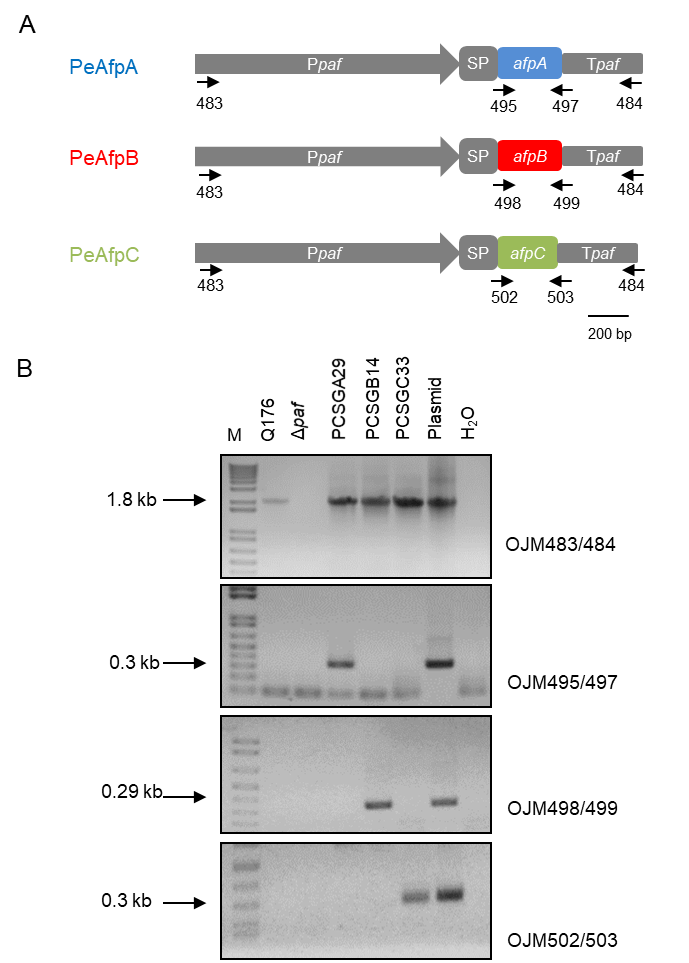


**Supplementary Figure 1.** **Molecular characterization of *P. chrysogenum* transformant strains for PeAFP production.** (A) Schematic representation of gene expression systems used to produce proteins PeAfpA (blue), PeAfpB (red) and PeAfpC (green) in *P. chrysogenum.* In grey: *paf* promoter (P*paf*), *paf* SP-pro sequence (SP), and *paf* terminator (T*paf*). Arrows show localization of primers used for PCR confirmation of the positive transformants for each gene construction. (B) PCR amplifications of genomic DNA for the confirmation of the different *P. chrysogenum* transformant strains, using the primer pairs indicated in the figure. The strains in the figure are: the PAF producer *P. chrysogenum* wild-type strain Q176, the null Δ*paf* strain used as recipient for PeAFP constructions, and *P. chrysogneum* transformant strains PCSGA29, PCSGB14 and PCSGC33, which produce PeAfpA, PeAfpB and PeAfpC, respectively.

**Supplementary Figure 2. MALDI-TOF MS analyses.** Isotopic average molecular mass (m/z) of the two AFPs from *P. expansum* PeAfpB (A) and PeAfpC (B) produced in *P. chrysogenum.*

**Supplementary Figure 3. Characterization of *P. expansum* class C AFP PeAfpC.** (A) Sequence alignment of Class C AFPs and AFP-like mature sequences. Conserved cysteine residues are highlighted in bold. The eleven extra amino acids predicted in PeAfpC but absent in the rest of class C homologs are highlighted in red. (B) Peptide Mass fingerprint (PMF) of pure PeAfpC. PMF revealed that PeAfpC lacks the predicted eleven amino acid insertion.

**Supplementary Figure 4. Analyses of supernatants of *P. chrysogenum* PeAFP producing strains by SDS-PAGE and Western blot.** SDS-PAGE of 10 µL of PcMM supernatants of *P. chrysogenum* transformant strains (top) ordered as follows: the parental strain Δ*paf,* PeAfpA producer strain PCSGA29, PeAfpB producer PCSGB14, and PeAfpC producer strain PCSGC33. Protein production was analized after 72 and 96 h of fungal growth. M: Seeblue® Pre-stained protein standard. Western blot confirmation of the proteins produced by *P. chrysogenum* transformants using specific antibodies against PeAfpA, PeAfpB and PeAfpC (bottom).

## Supplementary Tables
